# Trends in drug use and prescription patterns for Crohn’s disease patients in China, 2018–2022

**DOI:** 10.1186/s12913-026-14230-w

**Published:** 2026-02-26

**Authors:** Li Wang, Linpo Zhou, Yao Zhu, Mengdan Zhao, Fan Wu

**Affiliations:** 1 Department of Pharmacy, Women’s Hospital, Zhejiang University School of Medicine, Hangzhou, 310006 China; 2 Hospital of Zhejiang People’s Armed Police, Hangzhou, 314000 China; 3 Department of Pharmacy, The First Affiliated Hospital of Zhejiang Chinese Medical University (Zhejiang Provincial Hospital of Chinese Medicine), Hangzhou, 310006 China

**Keywords:** Crohn’s disease, Prescription, China, Infliximab, Pharmacoepidemiology

## Abstract

**Background:**

Crohn’s disease (CD) is an inflammatory bowel disease that requires medical treatment. The use of medications to treat CD is increasing worldwide, coinciding with the rising prevalence of the disease. However, research on the use of medications for CD has mostly focused on developed countries, and little research has been done on the status and trends in China. Thus, we conducted a nationwide study to ascertain the current status of treatment for CD patients in Chinese healthcare institutions.

**Methods:**

Data on inpatient prescriptions for CD were obtained from the Hospital Prescription Analysis Cooperative Project of China. We analyzed the trends in patient numbers and prescription expenditures in seven regions of China. Subgroup analyses were performed by age, gender and drug class.

**Results:**

A total of 71,654,361 patients from 70 hospitals in seven major cities of China were included in the study. From 2018 to 2022, the proportion of patients with CD increased from 0.23 per 1000 patients to 0.40 per 1000 patients. Visits by CD patients increased from 3598 to 5389, and the relevant expenditure increased from 2.83 million Chinese Yuan in to 10.61 million Chinese Yuan. The 20–39 age group was the most predominant age of incidence. The prevalence in males was more than twice as high as in females. 5-aminosalicylic acid, enteral/parenteral nutrients, corticosteroids and biologicals were the main prescribed drugs. There was a clear upward trend in the use of biologicals, especially infliximab, which has become the most used prescription drug since 2020.

**Conclusions:**

This nationwide study indicates a significant increase in healthcare utilization and expenditures for CD in China from 2018 to 2022, concurrent with a paradigm shift in treatment from traditional therapies toward biologic agents, primarily infliximab. This shift, facilitated by national insurance coverage, underscores the need for pharmacoeconomic evaluations and optimized stewardship of advanced therapies. The findings establish a crucial baseline for informing future healthcare strategies for CD management in China.

## Background

Crohn’s disease (CD) is a chronic inflammatory disease of the gastrointestinal tract characterized by segmental, asymmetric and transmural inflammation. Anywhere of the gastrointestinal tract can be affected, with the terminal ileum and colon being the most commonly affected [1, 2]. The primary symptoms include abdominal pain, diarrhea, and weight loss. Additionally, many patients experience both intestinal and extraintestinal complications [3]. CD has emerged as a global public health challenge while the incidence of CD differs by region. In general, CD is more common in developed countries than in developing countries. For example, the highest prevalence values reported were in Europe (Germany [4], 322 per 100,000 people) and North America (Canada [5], 319 per 100,000 people). At the turn of the twenty-first century, the incidence of CD has been increasing in newly industrialized countries in Africa, Asia, and South America [6, 7]. From 2001 to 2015, CD prevalence in Taiwan rose from 0.6 to 3.9 cases per 100,000 people. The prevalence of CD in Hong Kong was 18.6 cases per 100,000 people in 2014 [8, 9]. However, there is still a lack of national studies of patients with CD in mainland China.

The pathogenesis of CD involves a multifactorial combination of genetic susceptibility, environmental factors, microorganisms and immunity. Medication is the mainstay of treatment for patients with CD, except in specific cases (refractory patients, or if medication is not tolerated) where surgery is the treatment of choice. For many decades, corticosteroids, aminosalicylates, and immunosuppressants have been the mainstay of CD management. However, in recent decades, a new chapter in the treatment of CD has opened up with the emergence of new biologicals. Anti-TNF therapies (such as infliximab and adalimumab) are effective to induce and maintain remission in CD [10]. Some newer biologicals, including ustekinumab and vedolizumab, have been approved in multiple countries for treating moderate-to-severe CD and have shown efficacy [11, 12]. Various factors influence the choice of medication, including disease severity, response to previous treatments, safety, guidelines, the physicians’ experience, cost and so on. In China, the majority of patients with CD are covered by the national basic medical insurance system. Medications, including conventional therapies and an increasing number of biologic agents, are reimbursed according to the National Reimbursement Drug List (NRDL), with patients responsible for a co-payment. The clinical setting for administration also varies; for instance, intravenous biologics such as infliximab are predominantly administered in hospital day-care or inpatient units, whereas subcutaneous agents and oral medications can be managed in outpatient clinics.

Currently, there is limited research on medication use in Chinese patients with CD. No studies have reported regional differences in CD in China. Thus, to assess the temporal trends and patterns of prescriptions for patients with CD in China, we conducted a cross-sectional study in seven major regions of China from 2018 to 2022.

## Methods

### Study design

This retrospective research study was designed based on prescription data. Ethical approval for this study was obtained from the First Affiliated Hospital of the Zhejiang Chinese Medical University. Due to the nature of the study, there was no need for informed consent as part of the approval process.

### Data source

Prescribing information was obtained from the Hospital Prescription Analysis Cooperative Project database, a large-scale, nationwide prescription cooperation program that is widely used and methodologically established for pharmacoepidemiologic studies in China. The database employs a standardized protocol in which participating hospitals provide complete prescription records for 40 randomly selected days per year (10 days per quarter). This scientifically designed sampling strategy aims to capture representative prescribing patterns while controlling for seasonal and holiday-related variations, and has been successfully applied in multiple prior national drug utilization studies [13–15].

Each anonymized prescription record includes a unique prescription code, date, patient demographics (age, gender), diagnosis, prescribing department, drug name, dosage, and cost. Patient identifiers are not available; therefore, each record is treated as an independent prescription visit for the purpose of analyzing aggregate trends.

This study analyzed inpatient prescriptions issued on the sampling days that met the following criteria: (1) diagnosis of Crohn’s disease (regardless of disease location, severity, or comorbidities); (2) issued from hospitals in seven major Chinese cities—Shanghai, Guangzhou, Hangzhou, Chengdu, Zhengzhou, Shenyang, and Tianjin—that participated continuously from 2018 to 2022. These cities represent geographically and socio-economically diverse regional centers across China. All possess well-developed tertiary healthcare infrastructures and operate within the country’s largely unified national health insurance system, though minor local variations in reimbursement details may exist.

A total of 70 hospitals were included, comprising 64 tertiary and 6 secondary institutions. This sample provides broad geographic coverage and reflects prescribing practices within major healthcare settings across China, supporting the analysis of national-level trends in inpatient CD management.

### Data analysis

We analyzed the number and cost of prescriptions in the seven regions over the years and calculated the percentage of number and spent by patients with CD. Prescription information for CD patients was stratified by age, gender, and drug. The Cochran-Armitage trend test will be used to analyze trends in proportions; other trends will be analyzed by the Mann-Kendall trend test. The statistical analysis was performed using R Version 4.3.2 software from http://www.R-project.org. A *p*-value < 0.05 was considered statistically significant.

## Results

### Trends in patient numbers and prescription expenditures among inpatients in seven regions of China

**Fig. 1:**
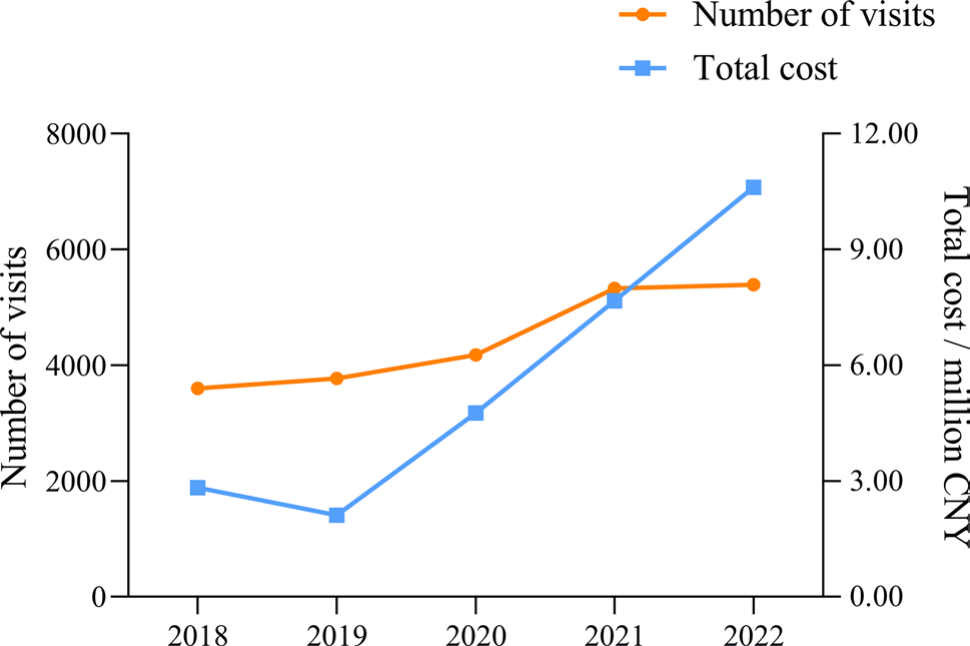
Trends in visits and cost among CD patients in 70 sample hospitals located in seven regions of China, 2018–2022

Over the past five years, a total of 71,654,361 patients were enrolled in our study. Table 1 shows that the number of visits did not exhibit a clear trend, decreasing from 155,998,815 in 2018 to 135,692,290 in 2022. However, the number of CD patients prescribed increased significantly over the study cycle (*p* = 0.03), from 3598 in 2018 to 5389 in 2022, the proportion of patients with CD also increased from 0.23 per 1000 patients in 2018 to 0.40 per 1000 patients in 2022 (*p* < 0.01). In terms of prescription value, the total cost in the seven regions of China did not show a significant trend from 2018 to 2022, while the prescription expenditure for CD patients increased from 2.83 million Chinese Yuan (CNY) in 2018 to 10.61 million CNY in 2022, and the proportion of expenditure increased from 0.06% in 2018 to 0.19% in 2022 (*p* < 0.01), which was shown in Table 1 and Fig. 1.

**Table 1:** Prescribing profiles of CD patients in seven regions of China, 2018–2022

	2018	2019	2020	2021	2022	**P**_**1**_
Number of CD patients prescribed	3598	3773	4177	5328	5389	0.03
Total number of patients prescribers	15599815	15699932	12716879	14068445	13569290	0.46
CD patient prescription amounts (million CNY)	2.83	2.11	4.77	7.67	10.61	0.09
Total prescription amounts (million CNY)	5118.86	5668.81	5174.71	5605.25	5543.55	0.81

Note: CNY, Chinese Yuan; P_1_, *p*-value for trend in number of patients and cost assessed by Mann–Kendall trend test

**Fig. 2:**
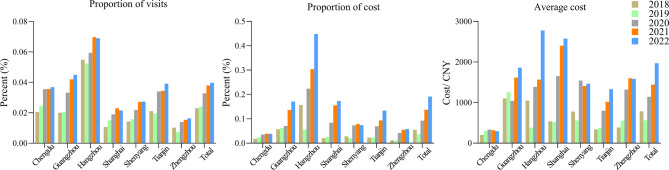
Trends in the proportion of visits for CD patients, the proportion of cost, and the average cost in seven regions of China, 2018–2022

Specifically for each region, as shown in Fig. 2, all seven regions showed a significant increasing trend in the percentage of CD patients prescribed (all *p* < 0.01). Hangzhou had the highest percentage of CD patients, with an average annual percentage of 0.61 per 1000, while Zhengzhou had the lowest percentage of CD patients, with an average annual percentage of 0.13 per 1000. All seven regions exhibited a significant increase in the percentage of prescription costs for CD patients during the study period (all *p* < 0.05). The smallest percentage of prescription expenditures was observed in Chengdu, with an annual average of 0.03%, while the largest was observed in Hangzhou, with an average of 0.24% per year. In addition, we calculated the average cost for CD patients by region and nationally. Overall, prescription spending increased nationwide from 786.74 million CNY in 2018 to 1969.37 million CNY in 2022 (*p* = 0.09). However, there were regional differences, with Shanghai, Hangzhou, and Guangzhou having higher average prescription costs than the rest regions of China, and Chengdu having the lowest average cost.

### Trends stratified by age and gender

**Fig. 3:**
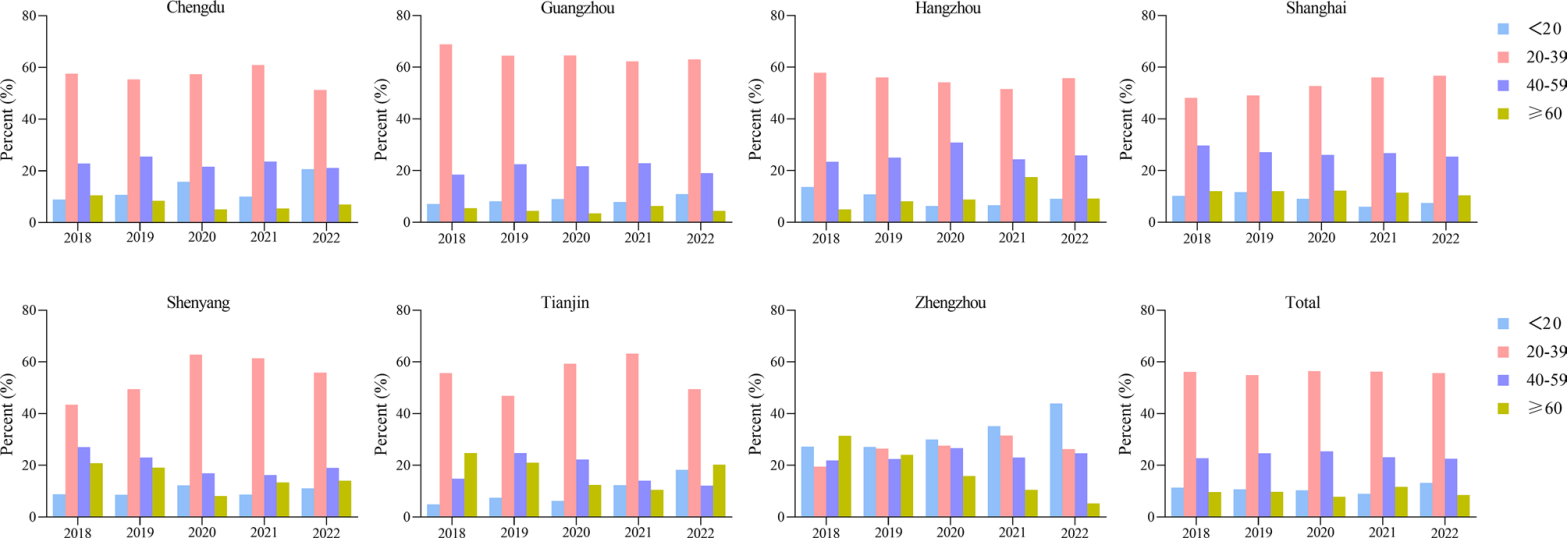
Trends in age distribution of CD patients in seven regions of China, 2018–2022

Figure 3 shows the age distribution of CD patients in the seven regions and nationally. All regions, except for Zhengzhou, had a majority of patients in the 20–39 age group, which accounted for approximately half of the total. The age distribution of CD patients in Zhengzhou is relatively even but shows a certain trend of youthfulness. Overall, the main age of onset for CD patients was the 20–39 and 40–59 age groups, as demonstrated in Table 2, the distribution by age group did not exhibit a significant trend during the study period (all *p* > 0.05).

**Table 2:** Age structure and gender distribution of CD patients, 2018–2022

		Number of patients (%)					**P**_**1**_	**P**_**2**_
		2018	2019	2020	2021	2022		
**Age** **(years)**	**<20**	409 (11.73)	403 (10.68)	434 (10.39)	482 (9.05)	711 (13.19)	0.09	0.15
	**20–39**	2021 (56.17)	2072 (54.92)	2356 (56.40)	2997 (56.25)	3003 (55.72)	0.03	0.90
	**40–59**	818 (22.73)	930 (24.65)	1059 (25.35)	1231 (23.10)	1215 (22.55)	0.09	0.31
	**≥60**	350 (9.73)	368 (9.75)	328 (7.85)	618 (11.60)	460 (8.54)	0.46	0.80
**Gender**	**Male**	2251 (62.56)	2518 (66.74)	2895 (69.31)	3687 (69.20)	3708 (68.81)	0.03	<0.01
	**Female**	1347 (37.44)	1255 (33.26)	1282 (30.69)	1641 (30.80)	1681 (31.19)	0.22	<0.01

P1, *p*-value for trend in number of patients, assessed by Mann–Kendall trend test; P2, *p*-value for trend in proportion of patients, assessed by Cochran-Armitage trendanalysis

**Fig. 4:**
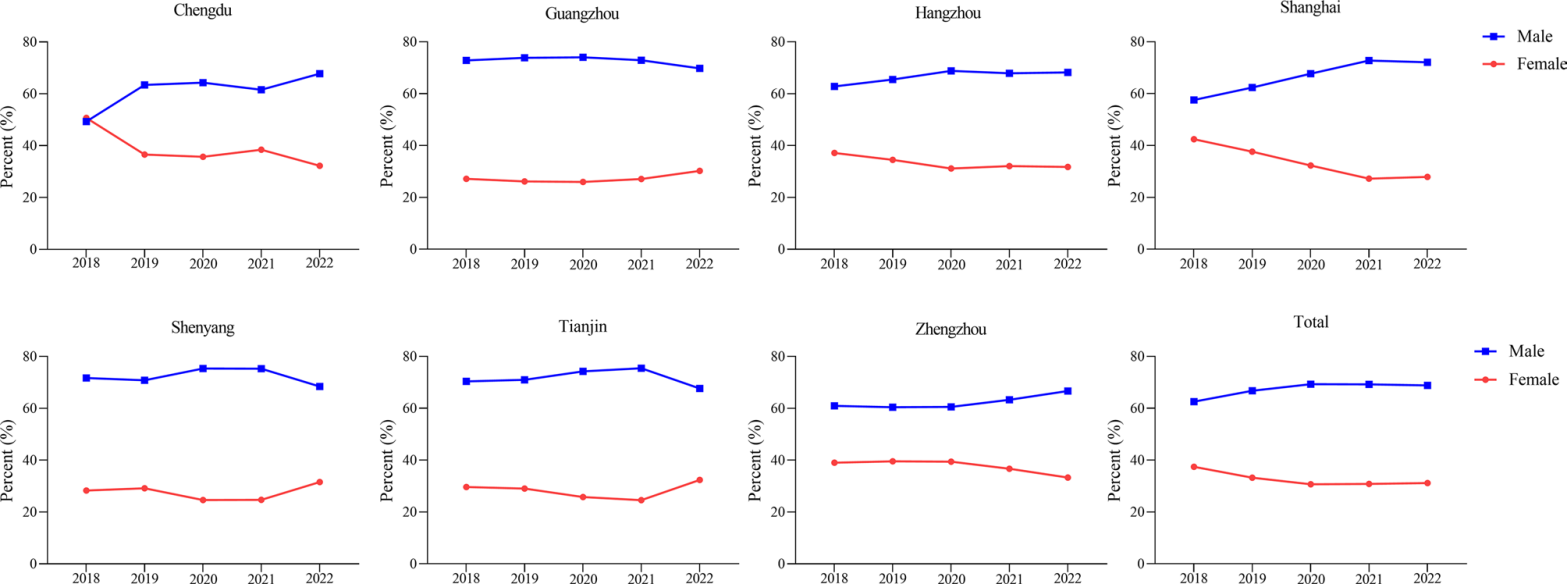
Trends in gender distribution of CD patients in seven regions of China, 2018–2022

In regards to gender distribution, all seven regions showed a similar pattern of more males than females, as shown in Fig. 4. In 2022, approximately 70% of patients were male, while around 30% were female. There is a noticeable trend towards a higher percentage of male patients over the study period (*p* < 0.01), shown in Table 2.

### Trends stratified by drug

**Fig. 5:**
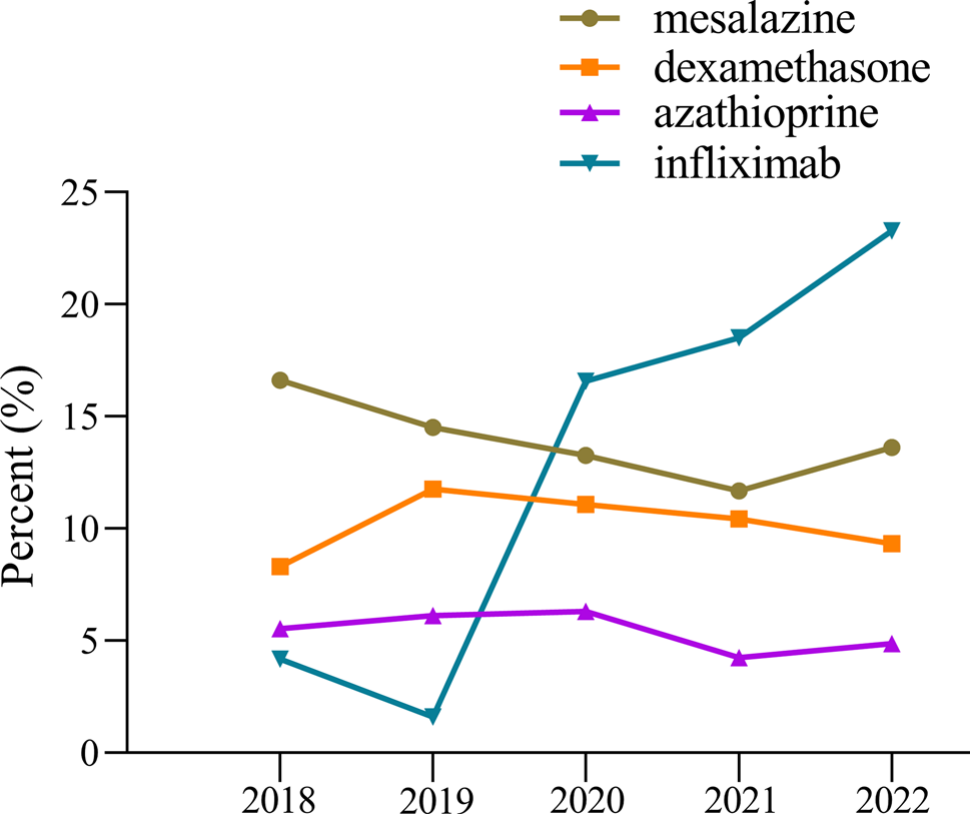
Trends in prescription utilization of several representative drugs, 2018–2022

The yearly prescriptions of each specific drug are shown in Tables 3 and Fig. 5. 5-aminosalicylic acid, enteral/parenteral nutrients, corticosteroids and biologicals were the main prescribed drugs. Mesalazine, a representative drug of the aminosalicylic acid class, has shown a significant downward trend in utilization (*p* < 0.01), although it remains at a high level. Infliximab was the most commonly prescribed biological for CD patients since 2020 and its usage was increasing significantly (*p* < 0.01), other biologicals also show an upward trend (all *p* < 0.01). Corticosteroids were predominantly treated with dexamethasone, which was used between 8.31% and 11.77% throughout the study period, indicating relative stability. The utilization of azathioprine decreased over time, and by the end of the study, its usage was less than 5%. The use rate of enteral nutritional powder was maintained at a level of approximately 14% throughout the cycle while the use of enteral nutrition preparations is declining year by year (all *p* < 0.01). As for gut microbiota drugs, the use of compound eosinophil-lactobacillus and bifid triple viable has decreased. The use of live combined Bacillus subtilis and Enterococcus has increased annually (all *p* < 0.01). Levofloxacin and metronidazole are commonly used antibiotics in CD patients, and their use decreased during the study period (all *p* < 0.01).

**Table 3:** Prescription visits by drug and drug class, 2018–2022

Drug class	Drug	Number of patients (%)					**P**_**1**_	**P**_**2**_
		2018	2019	2020	2021	2022		
5-aminosalicylic acid	mesalazine	598 (16.62)	547 (14.50)	554 (13.26)	622 (11.67)	734 (13.62)	0.22	<0.01
Corticosteroids	dexamethasone	299 (8.31)	444 (11.77)	463 (11.08)	555 (10.42)	502 (9.32)	0.09	0.95
Immunosuppressants	azathioprine	199 (5.53)	231 (6.12)	263 (6.30)	226 (4.24)	262 (4.86)	0.46	0.01
Biologicals	infliximab	150 (4.17)	60 (1.59)	692 (16.57)	986 (18.51)	1255 (23.29)	0.09	<0.01
	adalimumab	0 (0.00)	0 (0.00)	13 (0.31)	25 (0.47)	119 (2.21)	0.04	<0.01
	ustekinumab	0 (0.00)	0 (0.00)	0 (0.00)	9 (0.17)	212 (3.93)	0.10	<0.01
	vedolizumab	0 (0.00)	0 (0.00)	0 (0.00)	68 (1.28)	95 (1.76)	0.10	<0.01
Enteral/Parenteral nutrients	enteral nutritional powder (PT)	536 (14.90)	588 (15.58)	573 (13.72)	724 (13.59)	752 (13.95)	0.09	0.08
	glutamine (Gln)	592 (16.45)	695 (18.42)	681 (16.30)	587 (11.02)	552 (10.24)	0.22	<0.01
	compound amino acid 18AA-II	246 (6.84)	309 (8.19)	222 (5.31)	304 (5.71)	256 (4.75)	1.00	<0.01
	medium/long chain fat emulsion	161 (4.47)	161 (4.27)	182 (4.36)	216 (4.05)	161 (2.99)	0.58	<0.01
Gut microbiota drugs	compound eosinophil-lactobacillus	225 (6.25)	213 (5.65)	168 (4.02)	196 (3.68)	160 (2.97)	0.09	<0.01
	bifid triple viable	194 (5.39)	196 (5.19)	259 (6.20)	221 (4.15)	206 (3.82)	0.46	<0.01
	live combined Bacillus subtilis and Enterococcus	98 (2.72)	138 (3.66)	154 (3.69)	163 (3.06)	256 (4.75)	0.03	<0.01
Antibiotic	levofloxacin	212 (5.89)	241 (6.39)	217 (5.20)	228 (4.28)	217 (4.03)	1.00	<0.01
	metronidazole	182 (5.06)	203 (5.38)	226 (5.41)	215 (4.04)	217 (4.03)	0.22	<0.01

P1, *p*-value for trend in number of patients, assessed by Mann–Kendall trend test; P2, *p*-value for trend in proportion of patients, assessed by Cochran-Armitage trendanalysis

## Discussion

There is a scarcity of research on the prescription patterns of CD patients in non-Western countries, particularly in mainland China. Thus, we examined prescribing trends for patients with CD in seven regions of China from 2018 to 2022, based on a nationwide hospital prescribing database. To date, this is the largest pharmacoepidemiologic study of CD patients conducted in mainland China, and the results are nationally representative.

Due to the impact of COVID-19 that began to sweep the world in late 2019, there was a significant decline in both outpatient and inpatient volume across healthcare facilities at all levels in China [16, 17], which were also reflected in our findings, with varying degrees of abnormal fluctuations in both total patients and total prescription expenditures during 2020–2022. Nonetheless, we found a rapid upward trend in both the number and cost of CD patients over the study period, as well as the proportions. Some research findings suggest that CD may be associated with Western lifestyles, urbanization, and industrialization [18, 19]. Our study found that the percentage of CD patients was higher in Hangzhou and Guangzhou compared to other regions. Hangzhou and Guangzhou are both economically affluent and highly urbanized cities in eastern and southern China, respectively. Most studies have shown that the peak incidence of CD occurs in the second to fourth decade of life [20, 21], which is consistent with our results. However, unlike some other studies [1, 2, 20], we did not find an increasing trend in the percentage of older CD patients. Notably, we observed a marked increase in the proportion of patients aged < 20 years in 2022. This trend should be interpreted with caution, as it may reflect not only potential changes in disease epidemiology but also increased diagnostic awareness of pediatric CD, improved healthcare access for younger populations, or specific healthcare-seeking behaviors in the post-pandemic context. In contrast, the stable proportion of older patients throughout the study period may be explained by several factors. Diagnostic complexity in the elderly, where CD can mimic other gastrointestinal diseases, might lead to relative under-ascertainment in specialty care databases. Furthermore, treatment patterns for older adults may involve different risk-benefit considerations, potentially favoring outpatient management or therapies not fully captured in our inpatient prescription data. Remarkably, significant gender differences in CD were observed in our study. The ratio of males to females increased from 1.67 in 2018 to 2.21 in 2022, indicating a pronounced and growing male predominance in our cohort. This pattern is unlikely to be explained by differential access to healthcare or medication affordability, since China’s national insurance system provides uniform coverage, and overall prescription visits within the database are consistently higher among females. Instead, the observed sex distribution likely reflects true epidemiological characteristics of CD in this population. Although many studies have shown mixed results on sex-specific distribution in CD patients, with male-to-female ratios ranging from 0.59 to 2.94 [22–24], several studies of Asian populations have similarly reported a higher incidence of CD in males [25–28], supporting the possibility of regional or ethnic variation in disease expression. Such variability may be influenced by genetic background, environmental exposures, or sociobehavioral factors, including differences in occupational or educational access between sexes in certain settings.

The treatment of CD includes induction and maintenance therapy. The selection of a therapeutic drug is dependent on the severity of the disease and the response to the medication. 5-aminosalicylic acid, corticosteroids, immunosuppressants and biologicals are the most commonly used drugs for Crohn’s treatment. Mesalazine, an aminosalicylate representative, was approved by the FDA in 1987 for the treatment of CD and continues to be widely prescribed today. In an American database [29], 33.6% of patients with CD had a prescription for mesalazine during the period 2009–2014. However, there is no evidence to suggest that mesalazine is clinically or cost effective [30]. Therefore, recent ECCO and NICE guidelines no longer endorse 5-aminosalicylic acid for Crohn’s treatment [10, 31]. This shift is further supported by the 2021 American Gastroenterological Association guideline, which strongly recommends against the use of 5-ASA for CD treatment due to insufficient evidence of efficacy [32]. The decreasing trend observed in our study aligns with this global move towards evidence-based deprescribing of 5-ASA in CD. Mesalazine was also commonly used in our study but showed a significant decrease from 16.62% in 2018 to 13.62% in 2022. During the study period, dexamethasone was the most commonly used steroid in China. This differs from the prevalent use of prednisolone or methylprednisolone in Western countries, a pattern that may reflect historical prescribing habits, formulary availability, and cost considerations within the Chinese healthcare system. Nonetheless, as a potent systemic corticosteroid, it aligns with the guideline-recommended class effect for inducing remission in active CD.

Although systemic steroids are recommended to induce clinical response and maintain remission for patients with active, moderate-to-severe CD [31, 33], they are limited by their side effects and the risk of dependence. Furthermore, it is also important to note that prolonged use of steroids does not prevent disease recurrence [34]. Thus, we recommend an aggressive steroid-sparing strategy in cases of corticosteroid dependence or overdose. Immunosuppressants act by blocking lymphocyte proliferation, activation, or effector mechanisms. Thiopurines and methotrexate have shown efficacy in maintenance therapy in CD [35, 36]. There are several studies that suggest that thiopurines may reduce the risk of bowel resection [37, 38]. Azathioprine and 6-mercaptopurine are both thiopurine drugs and have similar efficacy. The choice between the two drugs is mainly based on medication habits, and in our country, physicians have more experience using azathioprine [39], which is consistent with the results of our study. Notably, an increased risk of malignancies [40–42], including lymphomas, non-melanoma skin cancers, and urothelial cancers, has been associated with the use of these drugs. For this reason, it is necessary for patients on long-term thiopurine therapy to receive regular monitoring. The observed low and decreasing use of azathioprine contrasts with higher utilization rates reported in some Western cohorts [43, 44]. This may reflect a more cautious approach towards thiopurine-related risks in China, a shift towards earlier biologic use facilitated by improved insurance coverage, and the inpatient setting of our data which may under-represent maintenance therapy. In general, switching to methotrexate may be considered for those who do not respond to or cannot tolerate thiopurine-based therapy. However, due to the limited data on methotrexate prescriptions in our study, we cannot elaborate on this.

Over the past twenty years, biological therapies have transformed the treatment of CD. ECCO recommends the use of TNF inhibitors, including infliximab, adalimumab and certolizumab pegol, to achieve remission in patients with moderate-to-severe CD who do not respond to conventional therapy [31]. In China, certolizumab pego is approved solely for the treatment of moderate to severe rheumatoid arthritis in adults and is not indicated for the treatment of CD. The NRDL included infliximab in November 2019 and adalimumab in January 2022, and our findings directly reflect the impact of this policy, as the use of these drugs showed a significant upward trend. These policy changes highlight how insurance reimbursement directly shapes prescribing patterns and facilitates access to advanced therapies, supporting a more top-down treatment approach. Infliximab is widely used globally and can be used as monotherapy or in combination with other therapies. Considering the potential immunogenicity of TNF inhibitors, the combination of immunomodulatory drugs may reduce the immunogenicity of CD patients for TNF-α [45, 46]. In patients with moderate to severe CD who have not responded adequately to conventional and anti-TNF therapy, the guidelines [10, 31] recommend the use of vedolizumab and ustekinumab for remission induction. Vedolizumab, a monoclonal IgG1 antibody that produces anti-inflammatory activity in the gut by blocking α4β7 integrins [47], entered the national medical insurance catalog in China on March 1, 2021. Ustekinumab is a fully human monoclonal antibody that blocks the p40 subunit of IL-12 and IL-23 [48]. It was added to the national medical insurance catalog in January 2022. Due to the short time since their entry into the Chinese market, the period of our study may not accurately reflect the trends in the use of these two drugs, and further follow-up studies are needed. The emergence of biologicals affects CD treatment strategies. In the past, patients with moderate-to-severe CD were typically treated using a step-up strategy, starting with aminosalicylates, cortisol or immunosuppressants and escalating to more effective therapies only after these treatments failed. However, this strategy has not effectively altered the course of the disease. Current opinion suggests that top-down therapeutic strategies, including early introduction of biologic therapy, should be considered for high-risk patients [49, 50]. Previous studies have shown that due to factors such as cost, health insurance policies and lack of experience among clinicians, biologicals are less commonly used in Asia than in Western countries [51, 52]. Our study showed a significant increase in the use of biologicals in China, from 4.17% in 2018 to 31.19% in 2022, reflecting a positive shift in clinical treatment strategies. This shift, particularly the rising use of intravenous infliximab which requires hospital-based administration, is likely an important contributor to the concurrent increase in inpatient visits observed in our study. While it reflects improved access to advanced therapy, it also underscores that the growing disease burden and evolving treatment paradigms together shape healthcare utilization for CD in China.

In this study, although the use of medications for nutritional support showed a downward trend in our study, they remain widely used. Malnutrition is common in patients with CD, nutritional supportive therapy should be given according to the condition and nutritional status, enteral nutrition is preferred, supplemented with parenteral nutrition in case of insufficiency [53]. The British Society of Gastroenterology recommends using complete enteral nutrition instead of steroids to induce remission in patients with mild to moderate CD [54]. Importantly, although complete enteral nutrition is recommended as first-line therapy to induce remission in pediatric Crohn’s patients [55], evidence for primary nutrition in adults is limited and requires further study [56]. Changes in the number and diversity of microbes in the gut are thought to be a key feature of Crohn’s disease [57]. So, modifying the gut microbiota through the administration of probiotics is a potential therapeutic approach for CD [58]. In addition to fecal microbiota transplantation, a more common clinical approach is oral gut microbial medications, with commonly used strains such as Bacillus licheniformis, Bifidobacterium bifidum, Enterococcus, Lactobacillus acidophilus. Our results show that live combined Bacillus subtilis and Enterococcus gradually replace other drugs as the most used gut microbial drug in China. Protection of intestinal integrity, regulation of epithelial cell proliferation, and remodeling of microbial structure and function may be among the mechanisms of action of Bacillus subtilis [59]. Despite the inconsistent efficacy of antibiotic regimens studied to date, antibiotics are still widely used to treat CD, especially perianal CD and septic complications [60]. Metronidazole and ciprofloxacin have been extensively researched. In our study, levofloxacin and metronidazole are the most commonly used antibiotics for clinical treatment of CD. Both ciprofloxacin and levofloxacin belong to the quinolone class of antibiotics, which may be selected based on drug resistance considerations.

This study has several limitations. First, regarding data representativeness, our sample was drawn predominantly from tertiary hospitals in major Chinese cities. Although this design enhances diagnostic accuracy and reflects prescribing practices in specialized care centers, it may not fully represent treatment patterns in secondary hospitals, rural settings, or outpatient clinics. Consequently, our findings may be more applicable to patients with moderate-to-severe disease typically managed in referral institutions. Second, concerning the nature of the data source, the database contains anonymized prescription visit records rather than longitudinally linked patient data. This prevents us from tracking individual treatment courses over time or analyzing clinical outcomes. Crucially, the study was limited to inpatient prescriptions; data on outpatient management, which represents a substantial component of CD care, were entirely absent. Third, methodological constraints should be noted. The use of 40 random sampling days per year operates under the assumption of consistent service availability across weekdays. While this standardized protocol is designed to capture seasonal trends, it may not fully account for very short-term prescribing variations. Furthermore, the database does not include clinical metrics such as disease severity, activity, or treatment indication, limiting our ability to assess the appropriateness of prescribed therapies. Our analysis also focused on overall trends; more granular subgroup analyzes were not conducted. Fourth, the potential influence of external factors should be considered. The observed trends, such as the marked increase in younger patients and in the use of biologics, may be influenced by concurrent changes in diagnostic awareness, updates in clinical guidelines, or the impact of the COVID-19 pandemic on healthcare-seeking behavior and drug availability. While we have acknowledged these contexts, our data cannot definitively disentangle their individual effects. Finally, as with all observational studies, unmeasured confounding factors (e.g., physician preference, nuanced local reimbursement policies, patient comorbidities) may influence prescribing patterns and cannot be fully adjusted for in our analysis.

## Conclusions

This nationwide analysis reveals a significant rise in healthcare utilization and economic burden due to CD in Chinese hospitals from 2018 to 2022. Prescription patterns demonstrate a clear paradigm shift, with biologics—led by infliximab—rapidly replacing traditional therapies. This transition, strongly aligned with guideline recommendations, was critically enabled by the inclusion of these agents in China’s National Reimbursement Drug List, improving patient access and likely contributing to increased inpatient visits.

Our findings highlight the urgent need for pharmacoeconomic evaluations to ensure the sustainable use of high-cost biologics, and identify opportunities to optimize care through better adherence to evidence-based guidelines. As the largest pharmacoepidemiologic study of inpatient CD management in mainland China, this work provides a crucial baseline for monitoring trends and informs future strategies for managing the growing burden of this disease.

## Data Availability

Data is provided within the manuscript or supplementary information files.
